# Supplementary material for: The Effect of Substrate Properties on Cellular Behavior and Nanoparticle Uptake in Human Fibroblasts and Epithelial Cells
Source: Nanomaterials (Basel). 2024 Feb 10;14(4):342. doi: 10.3390/nano14040342 (PMC10892529; doi:10.3390/nano14040342)
Supplement: Supplementary file 1 [file nanomaterials-14-00342-s001.zip › nanomaterials-2838058-supplementary.pdf]

Supporting Information

Effect of substrate properties on cellular behavior and nanoparticle uptake in human fibroblasts and epithelial cells

Mauro Sousa de Almeida [1], Aaron Lee [1,2], Fabian Itel [3], Katharina Maniura-Weber [4], Alke Petri-Fink [1,5] and Barbara Rothen-Rutishauser [1,*]

[1] Adolphe Merkle Institute and National Center of Competence in Research Bio-Inspired Materials,
University of Fribourg, Chemin des Verdiers 4, 1700 Fribourg, Switzerland; mauro.sousadealmeida@unifr.ch (M.S.d.A.); a.lee22@imperial.ac.uk (A.L.); alke.fink@unifr.ch (A.P.-F.)

[2] Department of Bioengineering, Imperial College London, South Kensington, London SW7 2BP, UK

[3] Empa, Swiss Federal Laboratories for Materials Science and Technology, Laboratory for Biomimetic
Membranes and Textiles, Lerchenfeldstrasse 5, 9014 St. Gallen, Switzerland; fabian.itel@empa.ch

[4] Empa, Swiss Federal Laboratories for Materials Science and Technology, Laboratory for Biointerfaces, Lerchenfeldstrasse 5, 9014 St. Gallen, Switzerland; katharina.maniura@empa.ch

[5] Department of Chemistry, University of Fribourg, Chemin du Musée 9, 1700 Fribourg, Switzerland

* Correspondence: barbara.rothen@unifr.ch

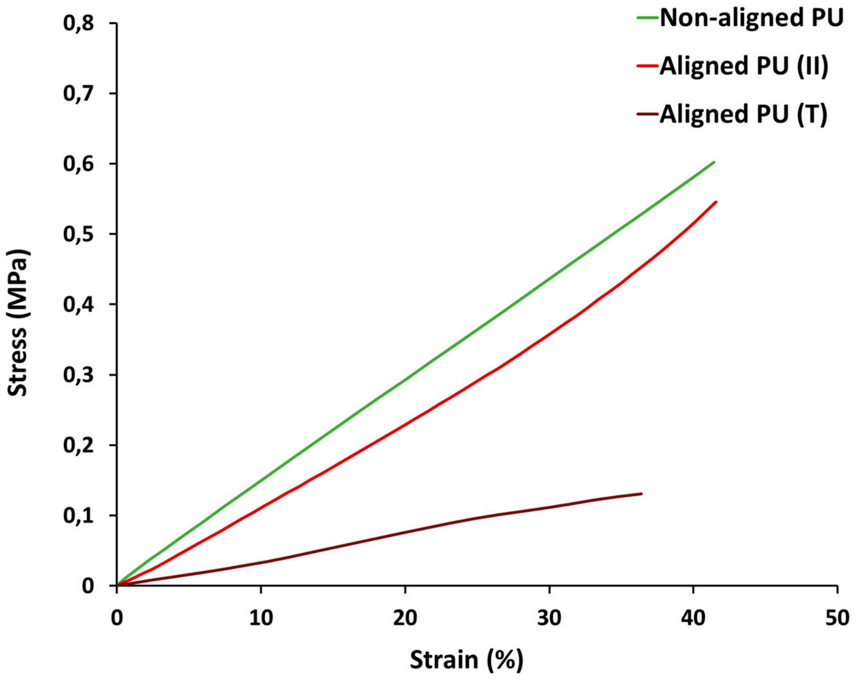

Figure S1 – Tensile test measurements of both aligned and non-aligned polyurethane (PU) fibers. II – fibers along the strain direction; T – fibers perpendicular to the strain direction.

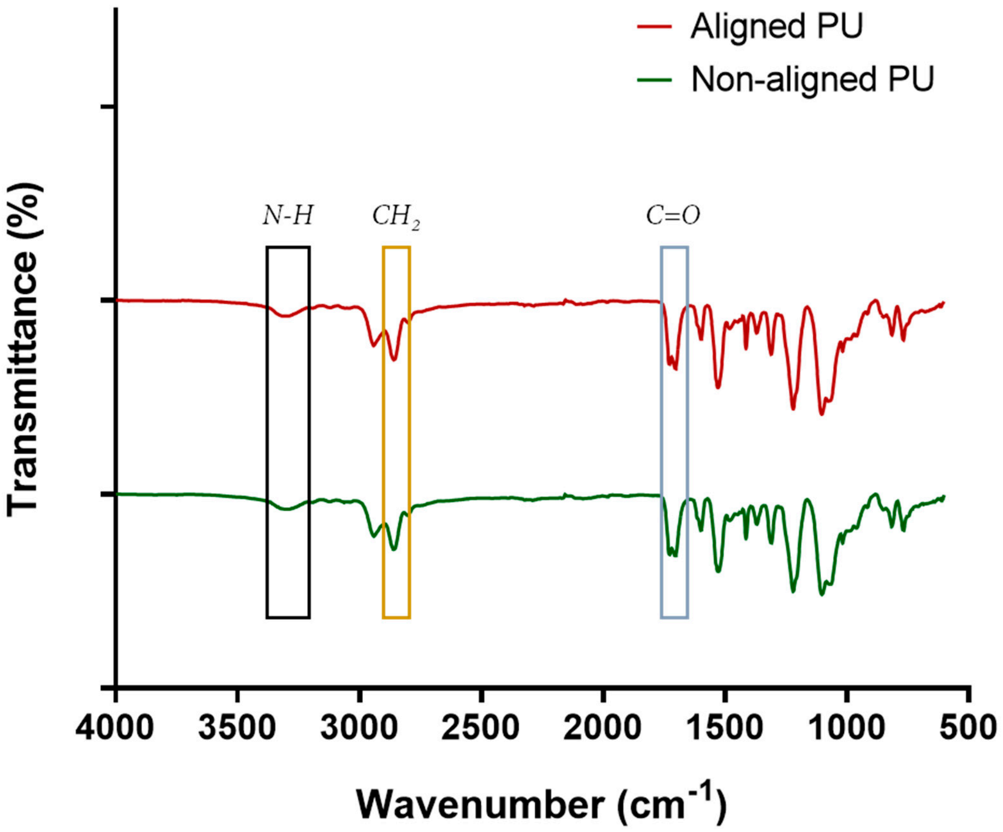

Figure S2 - Fourier-transformed infrared (FTIR) spectra of both aligned and non-aligned polyurethane (PU) fibers.

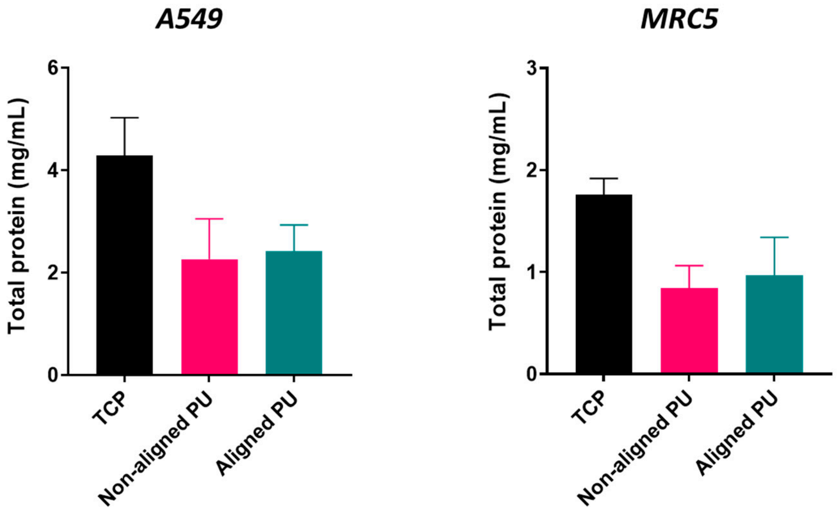

Figure S3 – Total protein content of A549 and MRC-5 cells after 72 and 48 hours, respectively. Data are presented as mean ± standard error of the mean (n=3).

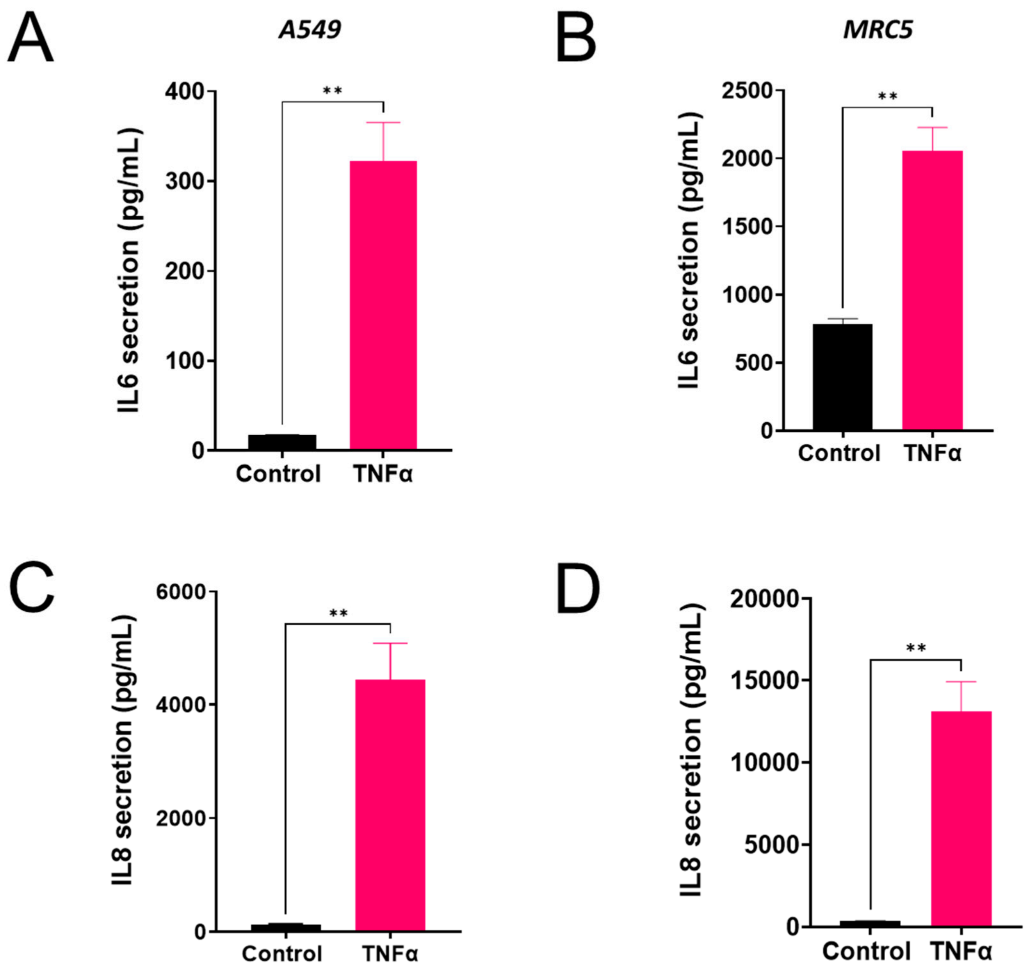

Figure S4 – Effect of the positive control tumor necrosis factor alpha (TNFα) on the secretion of inflammatory cytokines in lung epithelial cells (A549) and lung fibroblast (MRC-5). Bar graphs, at the top, show the release of interleukin (IL)-6 upon A549 (**A**) and MRC-5 (**B**) cell exposure to TNFα. Bar graphs, at the bottom, show the release of IL-8 upon A549 (**C**) and MRC-5 (**D**) cell exposure to TNFα. TNFα was added for 24 h at 1 µg/mL to A549 cells and 10 ng/mL to MRC-5 cells. Data are presented as mean ± standard error of the mean (n=3). Statistical significance was determined by unpaired t-test and represented by $^{**}p \leq 0.01$.

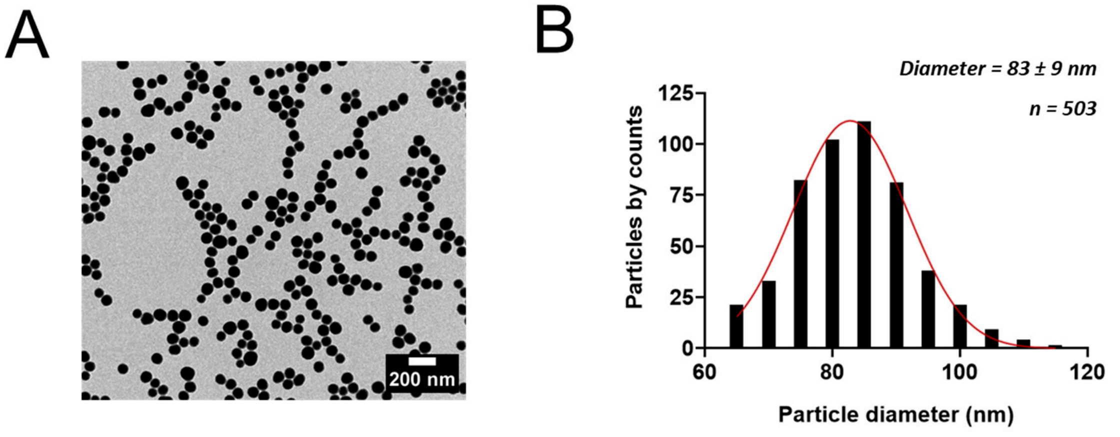

C

SiO$_2$-Cy5 80 nm	
Hydrodynamic diameter	99 nm
PDI	0.04
Zeta potential	- 46 ± 3 mV

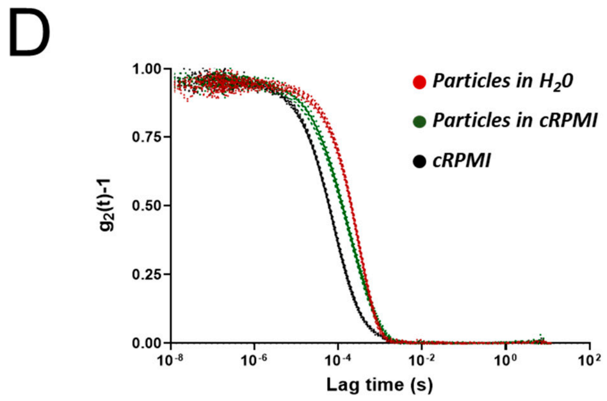

Figure S5 - Physicochemical properties of SiO_2 nanoparticles (NPs) and colloidal stability in cell culture medium. (**A**) Representative transmission electron microscopy (TEM) images and particle size distribution (**B**) of 83 nm SiO_2-Cy5 NPs. (**C**) Hydrodynamic diameter, polydispersity index (PDI) and zeta potential determined by dynamic and phase analysis light scattering in water. (**D**) Correlation functions of SiO_2-Cy5 NPs in water and supplemented cell culture medium (cRPMI) determined by dynamic light scattering. Correlation function of cRPMI only is also represented.

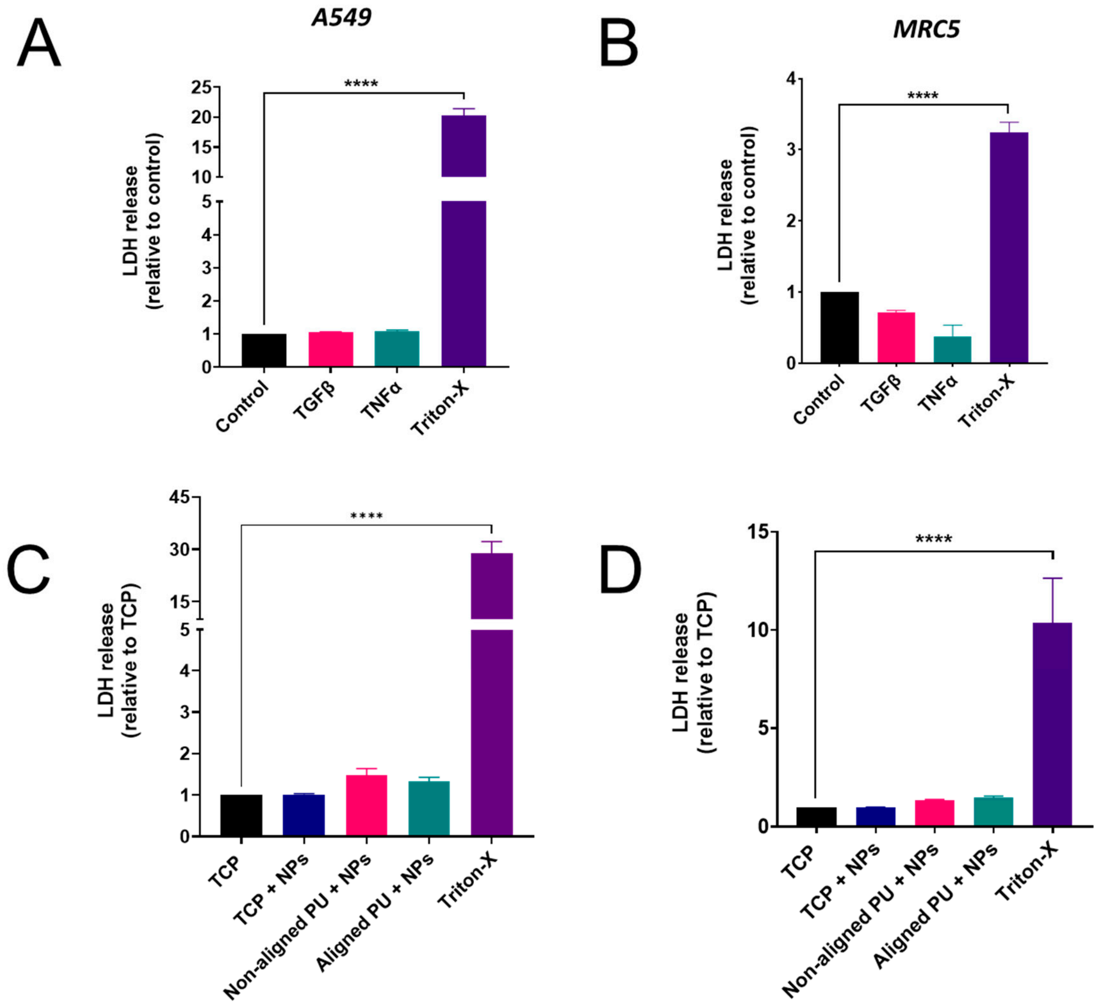

Figure S6 – Cytotoxicity of positive controls and SiO_2 nanoparticles (NPs) in lung epithelial cells (A549) and fibroblasts (MRC-5). Bar graphs, at the top, represent the release of lactate dehydrogenase relative to tissue culture plastic (TCP) from A549 (**A**) and MRC-5 cells (**B**) exposed to transforming growth factor beta (TGF-β) and tumor necrosis factor alpha (TNFα). TGF-β was added for 24 h to A549 and MRC-5 cells at a concentration of 10 ng/mL and 5 ng/mL respectively. TNFα was added for 24 h at 1 µg/mL to A549 cells and 10 ng/mL to MRC-5 cells. Bar graphs, at the bottom, show the release of lactate dehydrogenase relative to tissue culture plastic (TCP) from A549 (**C**) and MRC-5 cells (**D**) exposed for 24 h to 20 µg/mL of SiO_2 NPs. Data are presented as mean ± standard error of the mean (n=3). Statistical significance was determined by One-way ANOVA Dunnett's post-hoc test for multiple comparisons and represented by ****$p \leq 0.0001$.
